# Sickness Absence and Disability Pension in the Trade and Retail Industry: A Prospective Cohort Study of 192,000 White-Collar Workers in Sweden

**DOI:** 10.1097/JOM.0000000000002634

**Published:** 2022-07-29

**Authors:** Kristin Farrants, Kristina Alexanderson

**Affiliations:** From the Division of Insurance Medicine, Department of Clinical Neuroscience, Karolinska Institutet, Stockholm, Sweden (Farrants, Alexanderson).

**Keywords:** sick leave, sickness absence, disability pension, register study, occupational epidemiology, longitudinal follow-up, mental diagnoses

## Abstract

This study aimed to investigate future sickness absence (SA) and disability pension (DP) among privately employed white-collar employees in the trade and retail industry.

**Methods:**

A prospective population-based cohort study of all 192,077 such workers in Sweden in 2012, using linked microdata from nationwide registers, was conducted. Descriptive statistics of annual SA/DP during 2010 to 2016 and logistic regression for SA/DP in 2016 were used.

**Results:**

Women had more mean SA/DP net days/person; however, there were no sex differences in the mean number of net days/person with SA/DP. The mean number of net days/person increased, especially with mental diagnoses. Sickness absence in 2012 was the strongest factor associated with SA/DP in 2016 (women: odds ratio, 3.28; 95% confidence interval, 3.09–3.47; men: odds ratio, 4.10; 95% confidence interval, 3.76–4.48). Work-related factors were weakly associated with future SA/DP.

**Conclusions:**

The number of SA/DP net days per person increased, especially SA/DP days due to mental diagnoses.

## Abstract 2

White collar workers in the trade and retail industry have relatively low sickness absence/disability pension rates, although the rates increased over the study period, especially with mental diagnoses. Several factors were more strongly associated with SA/DP among men, indicating that there are other factors of importance for women.

Sickness absence (SA) and disability pension (DP) have consequences for society, for insurance agencies, for employers, and for the individual, in terms of financial losses, productivity losses, and a higher risk for future SA/DP.^1,2^

There is very little research about SA/DP among white-collar workers in the trade and retail industry, even though the trade and retail industry employs about 10% of those in activity (ie, employed, self-employed, or studying) in Sweden.^3^ Aside from one study on all privately employed white-collar workers in Sweden,^4^ most research on white-collar workers have used the Whitehall-II study of civil servants, of which the majority are white-collar workers,^5–7^ and the Helsinki Health Study of municipal workers, which includes both white-collar and blue-collar workers,^8,9^ both of which only include public employees. These studies have found differences in SA by sociodemographic and socioeconomic factors among white-collar workers.^6,10^ The aforementioned study on privately employed white-collar workers in Sweden also found differences by sociodemographic factors and branch of industry.^4^

Very little is known about SA in the trade and retail industry, despite its size.^11^ The trade and retail industry in general has relatively low rates of SA compared with other branches of industry.^12^ However, white-collar workers in the trade and retail industry comprise a range of different jobs, from executives and managers, to call-center operatives and administrative staff, and it is quite likely that the average numbers hide a large heterogeneity.

Some previous studies have found that SA rates are generally lower among white-collar workers than blue-collar workers.^10,13^ Although previous research has to a large extent focused on occupational groups with high SA rates, occupational groups with lower rates make up a substantial part of the labor market, and their SA has great implications for their companies, society, and themselves.^4^ In many countries, women have higher SA rates than men.^14^ Studies on the entire population have found differences in SA/DP by job-related factors such as job demands/job control^15–21^ and the size of the workplace.^22^ However, the extent to which this is also the case for privately employed white-collar workers is still unknown.

Currently, more than 100 different measures of SA are used in the literature.^23–25^ These mirror the challenges of SA research, such as skewed distributions of both the incidence and duration of SA, that many people have recurring events, that SA spells can be of different durations and grade, and that both incidence and duration matters, among others. The different measures use both different numerators (spells, days, individuals, etc) and different denominators (individuals at work, insured individuals, total individuals in the population etc). Different measures will lead to different results in the same data, for example, regarding gender differences in SA.^4,26^ Therefore, it is important to use several measures in studies of SA/DP.

The aim was to investigate future SA and/or DP in a cohort of white-collar employees in the trade and retail industry.

## METHODS

This is a population-based prospective cohort study of SA/DP in 2010 to 2016 among the white-collar workers who in 2012 were aged 18 to 67 years and privately employed in the trade and retail industry, using different measures of their SA/DP.

### Data and Study Population

We used microdata from 3 nationwide Swedish administrative registers, linked at individual level by the use of the personal identity number (a unique 10-digit number assigned to all residents in Sweden): (1) Longitudinal Integration Database for Health Insurance and Labour Market Studies held by Statistics Sweden; (2) MicroData for Analysis of the Social Insurance database held by the Social Insurance Agency; and (3) the Cause of Death Register held by the National Board of Health and Welfare.

The study population was all who were aged 18 to 67 years and registered as living in Sweden in both 2011 and 2012, had an occupational code according to the Swedish Standard for Occupational Classification that indicated a white-collar occupation, were employed at a private sector company in the trade and retail industry according to the Swedish Standard Industrial Classification, and during 2012 had income from work, parental benefits, and/or SA/DP that amounted to at least 7920 SEK (75% of the necessary income level to qualify for SA benefits from the Social Insurance Agency). The limit of 75% of the minimum income to qualify for SA benefits was set because, in many cases, SA benefits cover about 75% of the work income; without this adjustment, people with low incomes and long-term SA might have fallen below the minimum income level to be included in the study.^27^ Those who had full-time DP all of 2012 were excluded, whereas those who had SA or partial DP were included. This gave a cohort of 192,077 individuals.

In all analyses, individuals were excluded from the year after they emigrated or died, and they were also excluded from the analyses for a year if they did not have income from work, parental leave benefits, or SA/DP that year that exceeded 75% of the minimum income needed to qualify for SA benefits.

### Variables

We used information on the following variables from 2012 unless otherwise noted: sex: woman or man; age: 18 to 24, 25 to 34, 35 to 44, 45 to 54, 55 to 64, or 65 to 67 years; country of birth: Sweden, other Nordic country, other EU25, or rest of world including missing; educational level: compulsory school (≤9 years or missing), high school (10–12 years), or college/university (≥13 years); family situation: married/cohabiting with children at home, married cohabiting without children at home, single with children at home, or single without children at home; and type of living area: large city (Stockholm, Gothenburg, or Malmö), medium-sized town (>90,000 inhabitants within 30 km of city center), or small town/rural (<90,000 inhabitants within 30 km of city center).

For information on job demands/job control, we used a psychosocial job exposure matrix^28^ (for more details, see Ref. 29). We categorized individuals into 9 groups: high demands/high control, high demands/medium control, high demands/low control, medium demands/high control, medium demands/medium control, medium demands/low control, low demands/high control, low demands/medium control, and low demands/low control. Workplace size was categorized into 1 to 9 employees, 10 to 49 employees, 50 to 99 employees, 100 to 499 employees, and ≥500 employees.

Branch of industry in 2016 based on Swedish Standard Industrial Classification was categorized as follows: trade and retail, manufacturing, services, transport, construction and installation, care and education, or restaurants and hotels. Change of occupation between 2012 and 2016 based on Swedish Standard for Occupational Classification was categorized as follows: change within occupational category or no change, change of occupational category within the same major occupational group, change to a higher major occupational group (eg, from 2 to 1), change to a lower major occupational group (eg, from 1 to 2). Occupational sector in 2016 was categorized into municipal, region, state, private, or other.

Diagnosis-specific SA/DP was categorized into the following diagnosis groups: mental diagnoses (*International Classification of Diseases, Tenth Revision*, codes F00–F99 and Z73), musculoskeletal diagnoses (M00–M99), injuries (S00–T98 and V01–Y98), cancer (C00–D48), cardiovascular diagnoses (I00–I99), pregnancy-related diagnoses (among women: O00–O99 and N96), or other diagnoses (all other diagnoses, including missing).

### Measures

We calculated the following SA/DP measures:

- annual numbers and prevalence of people with SA/DP in 2010 to 2016
- annual mean number of SA/DP net days per person in 2010 to 2016
- annual mean number of SA/DP net days per person with SA/DP in 2010 to2016
- annual mean number of SA/DP net days in different diagnosis groups in 2010 to 2016
- odds ratios (ORs) for having SA/DP in 2016.

We also ran sensitivity analyses for the ORs of having SA/DP in 2016, excluding all those who had any SA or DP in 2012.

The project was approved by the Regional Ethical Review Board of Stockholm, Sweden.

### Public SA Insurance in Sweden

All people living in Sweden 16 years or older with an income from work or unemployment benefits are covered by the national public SA insurance and can claim SA benefits for a reduced work capacity due to disease or injury, without an upper age limit, although some restrictions to the length of SA apply after the age of 65 years. After a first qualifying day, the employer provides sick pay for days 2 to 14 of the SA spell, after which SA benefits are paid by the Social Insurance Agency. Those who are self-employed have more qualifying days. Those who are unemployed get SA benefits from the Social Insurance Agency after the first qualifying day. A physician certificate is required after 7 days of self-certification. In this study, data on SA with benefits from the Social Insurance Agency were used. Sickness absence spells ≤14 days were not included in the study, so as not to introduce bias regarding those who might have been unemployed. Sickness absence spells could be ongoing for years. All residents in Sweden aged 19 to 64 years, whose work capacity is permanently or long-term reduced because of disease or injury, can be granted DP from the Social Insurance Agency. Sickness absence benefits cover 80% of lost income, and DP benefits cover 64% of lost income, both up to a certain level.

Both SA and DP can be granted for part time or full time (25%, 50%, 75%, or 100% of ordinary work hours); this means that people can be on partial SA and DP at the same time. Therefore, we used net days so that partial days of SA/DP were combined. The number of net days were calculated using the number of gross days with benefits multiplied by the extent of absence (ie, 25%, 50%, 75%, or 100%), for example, 2 days of absence for 75% were counted as 1.5 net days.

## RESULTS

Table 1 shows the sociodemographic characteristics of the cohort in 2012. There was a slightly higher proportion of men (55.56%), and the vast majority were aged 25 to 64 years.

**TABLE 1:** Sociodemographic and Work-Related Information on the Study Cohort of Privately Employed White-Collar Workers at Baseline in 2012

		Total		Women		Men	
		n	%	n	%	n	%
All		192,077	100	85,356	100	106,721	100
Sex					
Women		85,356	44.44				
Men		106,721	55.56				
Age						
18–24 y		8145	4.24	4485	5.25	3660	3.43
25–34 y		40,881	21.28	20,277	23.76	20,604	19.31
35–44 y		60,739	31.62	26,864	31.47	33,875	31.74
45–54 y		50,160	26.11	20,759	24.32	29,401	27.55
55–64 y		29,607	15.41	11,986	14.04	17,621	16.51
65–67 y		2545	1.32	985	1.15	1560	1.46
Type of living area						
Large city		99,445	51.77	46,242	54.18	53,203	49.85
Medium-sized town		60,893	31.70	25,431	29.79	35,462	33.23
Small town or rural		31,739	16.52	13,683	16.03	18,056	16.92
Educational level, y						
Elementary (0–9 y)		14,612	7.61	4778	5.60	9834	9.21
High school (10–12 y)		99,554	51.83	42,259	49.51	57,295	53.69
University/college (>12 y)		77,911	40.56	38,319	44.89	39,592	37.10
Country of birth					
Sweden		175,508	91.37	76,709	89.87	98,799	92.58
Other Nordic country		4243	2.21	2324	2.72	1919	1.80
Other EU-25		3457	1.80	1685	1.97	1772	1.66
Rest of the world		8869	4.62	4638	5.43	4231	3.96
Family situation					
Married/cohabiting without children		25,595	13.33	11,344	13.29	14,251	13.35
Married/cohabiting with children		96,068	50.02	40,530	47.48	55,538	52.04
Single without children		58,416	30.41	25,457	29.82	32,959	30.88
Single with children		11,998	6.25	8025	9.4	3973	3.72
No. employees at workplace					
1–9		51,896	27.02	24,275	28.44	27,621	25.88
10–49		75,935	39.53	31,032	36.36	44,903	42.08
50–99		22,688	11.81	9626	11.28	13,062	12.24
100–499		32,752	17.05	15,948	18.68	16,804	15.75
≥500		8806	4.58	4475	5.24	4331	4.06
Job control/demands					
Low control, low demands		21,967	11.44	16,854	19.75	5113	4.79
Low control, medium demands		16,930	8.81	11,406	13.36	5524	5.18
Low control, high demands		25,128	13.08	21,968	25.74	3160	2.96
Medium control, Low demands		20,444	10.64	8894	10.42	11,550	10.82
Medium control, medium demands		21,919	11.41	6558	7.68	15,361	14.39
Medium control, high demands		21,663	11.28	12,486	14.63	9177	8.60
High control, low demands		21,614	11.25	2404	2.82	19,210	18.00
High control, medium demands		25,177	13.11	1641	1.92	23,536	22.05
High control, high demands		17,235	8.97	3145	3.68	14,090	13.20

The majority among both women and men lived in large cities (Stockholm, Gothenburg, or Malmö) and were born in Sweden. A very small proportion (5.60% of women and 9.21% of men) had only elementary education, whereas 44.89% of women and 37.10% of men had at least some college/university education. The majority were married/living with partner with children younger than 18 years living at home, but more than twice the proportion of women (9.40%) than men (3.72%) were single with children living at home.

Supplementary Figure 1, http://links.lww.com/JOM/B149, shows the distribution of job demands/control for all and for women and men in a kernel density plot. Among the entire population, the distribution seemed fairly even; however, although the level of demands was equally distributed among women and men, there were far more women in jobs with low control and far more men in jobs with high control.

**FIGURE 1:**
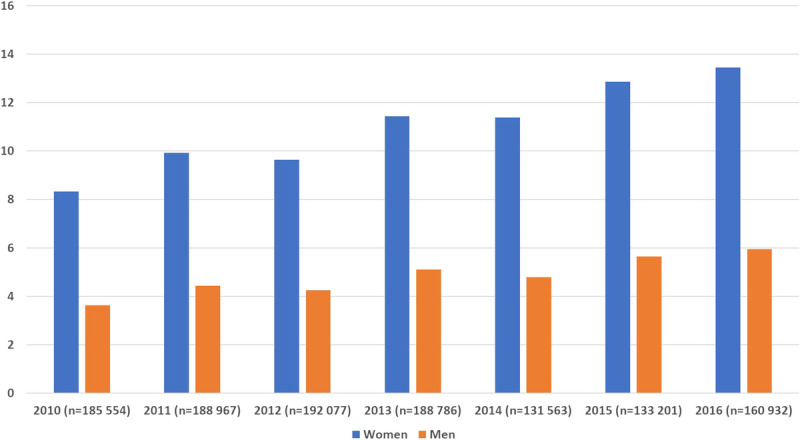
Proportions of white-collar workers in the trade and retail industry in the 2012 cohort who had any SA/DP, in each of the years 2010 to 2016, stratified by sex. DP, disability pension; SA, sickness absence.

Figure 1 shows the proportions of women and men who had at least some SA/DP during each of the study years 2010 to 2016. Each year, the proportions who had SA/DP were higher among women (8%–13%, depending on year) than men (3%–6% depending on year). The proportion of the cohort who had SA/DP increased slightly between 2010 (8% of women and 4% of men) and 2016 (15% of women and 6% of men).

**FIGURE 2:**
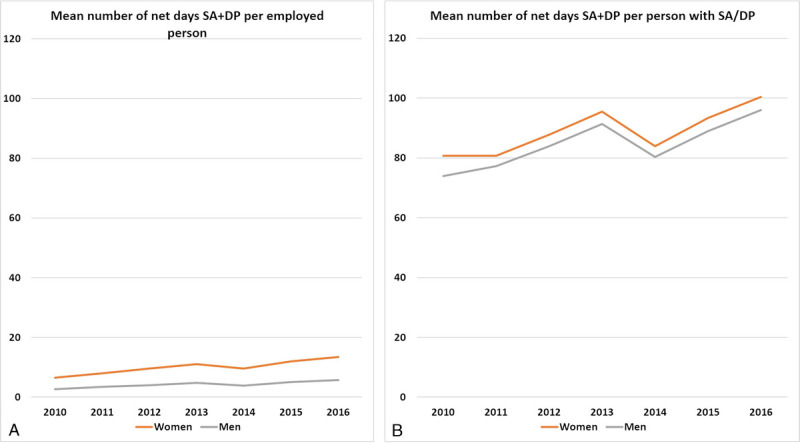
Mean number of SA and DP days per employed person and year (A) and per person with SA/DP in the respective year (B). DP, disability pension; SA, sickness absence.

Figure 2 shows the annual mean number of SA/DP days per person (A) and per person with SA/DP the respective year (B) during 2010 to 2016. The mean number of SA/DP days increased each year, from 6.6 mean days among women in 2010 to 13.5 in 2016 and from 2.7 to 5.7 among men, except for a slight dip in 2014 among both women and men. Women had more SA/DP days than men each year, and the increase in SA/DP days was slightly larger among women than men.

The annual mean number of SA/DP days per person with SA/DP was, as expected, much higher than the number of net days per employed person but also increased from 80.7 mean days among women in 2012 to 100.3 in 2016 and from 73.9 to 96.0 days among men, with a slight dip in 2014. There were larger sex differences in the mean number of SA/DP days per employed person than the mean number of SA/DP days per person with SA/DP.

**FIGURE 3:**
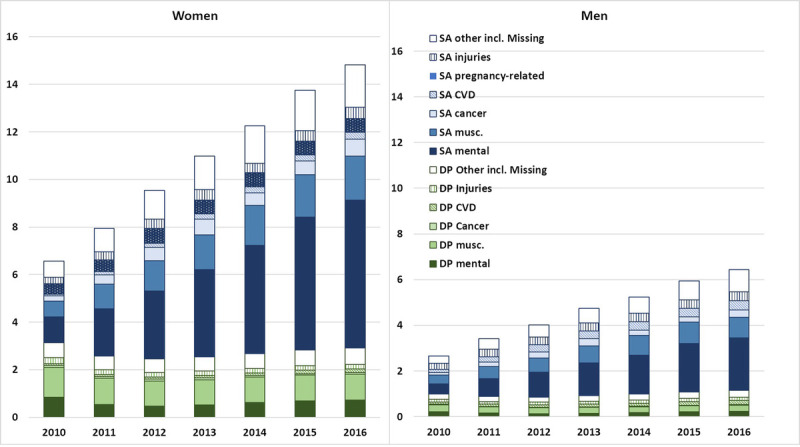
Mean number of net days with SA and DP, respectively, per person and year in different diagnosis groups. DP, disability pension; SA, sickness absence.

In Figure 3, the annual mean number of SA/DP days is presented by diagnosis groups. The mean number of days increased over the studied years among both women and men, especially days due to mental diagnoses. The increase in the other diagnosis groups was smaller, leading to mental diagnoses constituting an increasing proportion of SA/DP days.

Table 2 presents OR and 95% confidence intervals (CIs) for the risk of having SA/DP in 2016. Men had a lower risk than women of such SA/DP (OR, 0.46; 95% CI, 0.44–0.49). Those who were aged 65 to 67 years in 2012 were much less likely to have SA in 2016 (OR, 0.09; 95% CI, 0.05–0.18) than those aged 35 to 44 years. Otherwise, the ORs by age were relatively close to 1, rarely over 1.50 or under 0.67, with one exception: OR for men aged 55 to 64 years was 1.80 (95% CI, 1.64–1.97).

**TABLE 2:** Crude and Mutually Adjusted ORs and 95% CIs Over the Association Between Sociodemographic and Job-Related Factors in 2012 With SA and/or DP in 2016

	Total		Women		Men	
	Crude OR (95% CI)	Adjusted OR (95% CI)	Crude OR (95% CI)	Adjusted OR (95% CI)	Crude OR (95% CI)	Adjusted OR (95% CI)
All	192,077		85,356		106,721	
Sex						
Women	Ref	Ref				
Men	0.42 (0.40–0.43)	0.46 (0.44–0.49)				
Age						
18–24 y	0.94 (0.86–1.02)	0.84 (0.76–0.93)	0.84 (0.76–0.93)	0.85 (0.75–0.96)	0.86 (0.73–1.02)	0.76 (0.63–0.91)
25–34 y	1.04 (0.99–1.09)	1.01 (0.96–1.06)	1.03 (0.98–1.09)	1.05 (0.99–1.12)	0.89 (0.82–0.97)	0.88 (0.81–0.96)
35–44 y	Ref	Ref	Ref	Ref	Ref	Ref
45–54 y	1.22 (1.17–1.27)	1.19 (1.13–1.24)	1.18 (1.12–1.24)	1.09 (1.03–1.16)	1.38 (1.29–1.47)	1.36 (1.27–1.47)
55–64 y	1.11 (1.06–1.16)	1.28 (1.02–1.36)	0.91 (0.85–0.97)	0.97 (0.89–1.06)	1.55 (1.44–1.67)	1.80 (1.64–1.97)
65–67 y	0.04 (0.02–0.08)	0.09 (0.05–0.18)	0.04 (0.01–0.08)	0.07 (0.03–0.18)	0.06 (0.03–0.15)	0.14 (0.05–0.37)
Type of living area						
Large city (Stockholm, Gothenburg, Malmö)	Ref	Ref	Ref	Ref	Ref	Ref
Medium-sized town (>90,000 inhabitants)	1.10 (1.06–1.14)	1.07 (1.03–1.11)	1.14 (1.08–1.19)	1.07 (1.01–1.12)	1.19 (1.12–1.25)	1.07 (1.00–1.14)
Rural (<90,000 inhabitants)	1.27 (1.21–1.32)	1.15 (1.10–1.21)	1.27 (1.21–1.34)	1.15 (1.08–1.23)	1.37 (1.28–1.46)	1.14 (1.05–1.23)
Educational level						
Elementary (≤9 y)	1.51 (1.43–1.60)	1.64 (1.53–1.75)	1.54 (1.42–1.67)	1.54 (1.40–1.69)	2.20 (2.02–2.40)	1.83 (1.65–2.02)
High school (10–12 y)	1.30 (1.26–1.35)	1.29 (1.25–1.34)	1.28 (1.23–1.34)	1.22 (1.17–1.28)	1.66 (1.57–1.76)	1.51 (1.41–1.61)
University/college (>12 y)	Ref	Ref	Ref	Ref	Ref	Ref
Birth country						
Sweden	Ref	Ref	Ref	Ref	Ref	Ref
Other Nordic countries	1.24 (1.12–1.37)	1.01 (0.98–1.23)	1.06 (0.93–1.19)	1.08 (0.94–1.24)	1.30 (1.10–1.55)	1.16 (0.96–1.41)
Other EU25	0.99 (0.88–1.12)	0.98 (0.86–1.12)	0.96 (0.83–1.11)	1.01 (0.87–1.19)	0.9 (0.73–1.11)	0.94 (0.74–1.20)
Rest of the world	1.27 (1.19–1.36)	1.24 (1.15–1.34)	1.17 (1.08–1.28)	1.17 (1.07–1.29)	1.15 (1.02–1.3)	1.35 (1.18–1.54)
Family situation						
Married/cohabitant without children at home	Ref	Ref	Ref	Ref	Ref	Ref
Married/cohabitant with children at home	0.93 (0.89–0.98)	0.90 (0.85–0.96)	0.98 (0.92–1.05)	0.83 (0.77–0.90)	0.89 (0.83–0.96)	1.00 (0.91–1.10)
Single without children at home	1.04 (0.99–1.10)	1.06 (1.00–1.13)	1.06 (0.99–1.13)	1.00 (0.92–1.09)	1.02 (0.94–1.10)	1.17 (1.06–1.29)
Single with children at home	1.69 (1.58–1.81)	1.18 (1.10–1.28)	1.47 (1.36–1.60)	1.13 (1.02–1.24)	1.28 (1.12–1.46)	1.26 (1.08–1.46)
SA in 2012						
Yes	4.69 (4.50–4.90)	3.53 (3.37–3.71)	3.70 (3.51–3.90)	3.28 (3.09–3.47)	5.01 (4.65–5.41)	4.10 (3.76–4.48)
No	Ref	Ref	Ref	Ref	Ref	Ref
No. employees at workplace						
1–9	1.21 (1.17–1.26)	1.06 (1.01–1.10)	1.17 (1.11–1.22)	1.09 (1.03–1.15)	1.13 (1.06–1.20)	1.01 (0.94–1.08)
10–49	Ref	Ref	Ref	Ref	Ref	Ref
50–99	0.93 (0.88–0.98)	0.95 (0.90–1.01)	0.88 (0.82–0.94)	0.92 (0.85–0.99)	0.97 (0.89–1.05)	1.01 (0.92–1.10)
100–499	0.93 (0.89–0.98)	0.96 (0.91–1.01)	0.90 (0.85–0.95)	0.96 (0.90–1.03)	0.82 (0.76–0.89)	0.94 (0.86–1.02)
≥500	0.99 (0.92–1.07)	1.07 (0.98–1.16)	0.98 (0.89–1.08)	1.10 (1.00–1.22)	0.76 (0.65–0.88)	0.97 (0.83–1.13)
Control/demands						
Low control, low demands	1.50 (1.41–1.60)	1.08 (1.00–1.16)	1.04 (0.95–1.13)	1.05 (0.96–1.15)	1.02 (0.90–1.16)	1.10 (0.95–1.27)
Low control, medium demands	1.46 (1.36–1.56)	1.06 (0.99–1.14)	1.05 (0.96–1.15)	1.01 (0.92–1.11)	1.18 (1.05–1.33)	1.23 (1.07–1.40)
Low control, high demands	1.64 (1.55–1.75)	1.06 (0.99–1.14)	1.05 (0.97–1.14)	1.03 (0.94–1.12)	1.21 (1.04–1.40)	1.23 (1.05–1.45)
Medium control, low demands	1.05 (0.98–1.13)	0.96 (0.89–1.04)	0.91 (0.82–1.00)	0.93 (0.84–1.03)	0.97 (0.88–1.08)	1.01 (0.91–1.13)
Medium control, medium demands	Ref	Ref	Ref	Ref	Ref	Ref
Medium control, high demands	1.25 (1.17–1.34)	1.02 (0.95–1.09)	1.02 (0.93–1.12)	1.03 (0.93–1.13)	0.93 (0.83–1.04)	0.95 (0.85–1.07)
High control, low demands	0.78 (0.72–0.83)	0.94 (0.87–1.02)	0.95 (0.83–1.1)	0.99 (0.85–1.15)	0.93 (0.85–1.02)	0.90 (0.81–0.99)
High control, medium demands	0.71 (0.66–0.76)	0.90 (0.84–0.98)	0.83 (0.70–0.99)	0.85 (0.71–1.02)	0.92 (0.85–1.00)	0.88 (0.80–0.97)
High control, high demands	0.80 (0.74–0.86)	0.89 (0.81–0.96)	0.88 (0.77–1.01)	0.89 (0.77–1.02)	0.90 (0.81–0.99)	0.85 (0.76–0.95)
Job change						
Change within occupational category or no change (n = 93,999)	Ref	Ref	Ref	Ref	Ref	Ref
Change of occupational category within the same major occupational group (n = 43,372)	1.02 (0.99–1.07)	1.00 (0.96–1.04)	0.97 (0.92–1.02)	0.98 (0.93–1.03)	0.96 (0.9–1.03)	1.01 (0.94–1.08)
Change to a higher major occupational group (eg, from 2 to 1) (n = 19,568)	0.87 (0.82–0.92)	0.88 (0.82–0.93)	0.88 (0.82–0.94)	0.91 (0.85–0.98)	0.72 (0.65–0.8)	0.79 (0.71–0.87)
Change to a lower major occupational group (eg, from 1 to 2) (n = 32,382)	1.20 (1.15–1.25)	1.11 (1.06–1.16)	1.14 (1.08–1.21)	1.04 (0.97–1.10)	1.28 (1.2–1.37)	1.16 (1.08–1.25)
Branch of industry in 2016						
Construction (n = 2337)	1.00 (0.87–1.16)	1.05 (0.91–1.22)	0.83 (0.65–1.05)	0.74 (0.58–0.96)	1.43 (1.20–1.71)	1.34 (1.11–1.61)
Hospitality (n = 896)	1.32 (1.07–1.62)	1.14 (0.92–1.42)	1.06 (0.83–1.36)	1.01 (0.77–1.31)	1.46 (1.01–2.11)	1.56 (1.06–2.29)
Manufacturing (n = 9274)	0.83 (0.77–0.90)	0.93 (0.85–1.01)	0.86 (0.77–0.96)	0.90 (0.81–1.01)	0.93 (0.83–1.04)	0.97 (0.86–1.09)
Unknown (n = 10,676)	1.31 (1.24–1.40)	1.10 (0.88–1.38)	1.19 (1.10–1.29)	1.09 (0.82–1.44)	1.33 (1.21–1.45)	1.12 (0.78–1.62)
Services (n = 24,103)	0.90 (0.85–0.94)	0.89 (0.85–0.94)	0.88 (0.83–0.94)	0.87 (0.82–0.93)	0.85 (0.78–0.92)	0.93 (0.84–1.02)
Transport (n = 1217)	1.06 (0.87–1.28)	1.06 (0.87–1.30)	1.04 (0.79–1.36)	1.05 (0.80–1.39)	1.25 (0.95–1.64)	1.05 (0.78–1.41)
Care and education (n = 6252)	1.70 (1.58–1.83)	1.13 (1.02–1.26)	1.33 (1.23–1.45)	1.11 (0.99–1.25)	1.46 (1.23–1.73)	1.22 (0.97–1.52)
Trade and retail (n = 134,566)	Ref	Ref	Ref	Ref	Ref	Ref
Sector in 2016						
Municipal (n = 3880)	1.91 (1.75–2.09)	1.32 (1.17–1.48)	1.49 (1.35–1.65)	1.29 (1.13–1.47)	1.72 (1.41–2.10)	1.45 (1.14–1.84)
Region (n = 1146)	1.77 (1.50–2.09)	1.25 (1.03–1.51)	1.36 (1.13–1.62)	1.23 (1.00–1.52)	1.33 (0.85–2.08)	1.27 (0.79–2.07)
State (n = 5717)	1.63 (1.51–1.76)	1.27 (1.17–1.38)	1.28 (1.17–1.39)	1.26 (1.15–1.38)	1.23 (1.01–1.50)	1.33 (1.08–1.63)
Other (n = 3477)	1.41 (1.27–1.56)	1.24 (1.12–1.39)	1.30 (1.14–1.48)	1.25 (1.09–1.43)	1.35 (1.13–1.62)	1.25 (1.04–1.51)
Private sector (n = 158,476)	Ref	Ref	Ref	Ref	Ref	Ref

CI, confidence interval; DP, disability pension; OR, odds ratio; Ref, reference; SA, sickness absence.

Those with only elementary education had a higher risk of SA/DP than those with at least some university/college education, and this was stronger among men (OR, 1.83; 95% CI, 1.65–2.02) than among women (OR, 1.54; 95% CI, 1.40–1.69).

Having had SA in 2012 was associated with a much higher risk of having SA/DP in 2016, and again, this was stronger among men (OR, 4.10; 95% CI, 3.76–4.48) than among women (OR, 3.28; 95% CI, 3.09–3.47).

There were no large differences in the risk of SA/DP by the job-related factors in 2012 (size of company, job demands/control), nor by the job-related factors in 2016 (change of occupation, branch of industry, or sector); ORs were between 1.50 and 0.67. For men, there was a small but significant association between low control and a higher risk of SA/DP (ORs range, 1.10–1.23), whereas high control was associated with a lower risk (ORs range, 0.85–0.90), for all levels of job demands. However, for women, there were no such significant associations with job demands/control. Just over half the population did not change occupation or changed within the major occupational group (50.3%), 71% were still in the trade and retail industry, and 83% were still in the private sector at the end of follow-up.

Excluding those who had any SA/DP in 2012 did not change the magnitude of the estimates for the other variables in any major way (Supplementary Table 1, http://links.lww.com/JOM/B150).

## DISCUSSION

In this first exploratory prospective cohort study of SA/DP among all privately employed white-collar workers in the trade and retail industry in Sweden, we found that the rate of people with SA/DP was low and relatively stable over the years, whereas the mean number of SA/DP days increased slightly. The mean number of days per person with SA/DP increased especially, indicating that it was duration of SA/DP that increased rather than numbers of people on SA/DP. We also found that the mean number of SA/DP days due to mental diagnoses increased more than the mean number of such days due to other diagnoses. Although the women had slightly more SA/DP days per year than men in the entire cohort, there were no such sex differences in the mean number of SA/DP days among those who had SA/DP. That is, even though more women had SA/DP than men, there were no sex differences in length of SA/DP among those who had SA/DP.

Mental diagnoses were the most common SA/DP diagnoses, when somatic diagnoses were categorized in smaller groups. This has been found in several other studies of white-collar workers, although they have been either general studies among white-collar workers^4^ or of public employees, not specifically studied among people employed in the private trade and retail industry.^30^ Sickness absence spells due to mental diagnoses often become longer than SA spells due to other diagnoses in countries where long SA spells are possible, such as Sweden.^31,32^ Therefore, it is of interest to further study SA due to mental diagnoses in this occupational group. Which mental diagnoses are the most common, and which have the greatest risk of SA spells becoming long? Which interventions can prevent that such SA spells become long-term? To what extent do people on SA due to mental diagnoses later have SA due to other diagnoses and vice versa? How does the prevalence of SA due to mental diagnoses among white-collar workers in the trade and retail industry compare with white-collar workers in other branches of industry?

The mean number of SA/DP days per person with SA/DP increased more than both the proportion of individuals with SA/DP and slightly more than the mean number of net days of SA/DP per employed person. This indicates that the length or extent of SA/DP increased for those who were on SA/DP. The increase in SA/DP length could be related to the cohort getting older, as older age is associated with longer SA spells,^33^ and also to that SA spells due to mental disorders tend to be longer than spells due to other diagnoses.^31,32^ Compared with white-collar workers on the entire labor market, those in the trade and retail industry had slightly fewer SA/DP days, both among all and per person with SA/DP.^34^ That women have far more SA/DP days per person in total than men do, but a similar number of days per person with SA/DP, is in line with previous results on white-collar workers on the entire labor market.^4,34,35^

The strongest factors that predicted SA/DP in 2016 were SA in 2012, low educational level, and female sex. We found no strong associations between the included job-related factors (job demands/control, workplace size, change of occupation, branch of industry in 2016 or sector in 2016) and SA/DP in 2016. This indicates that there are other factors, either job-related, sociodemographic, socioeconomic, or morbidity-related, that explains the differences in SA/DP in this group, and this should be studied further. That job demands/control was not strongly associated with future SA/DP could possibly be due to the distribution of demands/control, which was much more even than in the entire population.^29^ We also found a much clearer differentiation by sex than previous studies have found in the general population, especially regarding the level of job control, where women were predominantly located in occupations with low control and men in occupations with high control. This is not as clear in studies of the entire population.^29,36^ The sex differences in the level of control could also possibly contribute to the sex differences in SA/DP; however, this needs to be investigated further. That change of occupation, branch of industry, or sector in 2016 was not strongly associated with SA/DP could be related to that most people were still in the same occupation, in the trade and retail industry and private sector. Changing your job can be a part of work rehabilitation, especially if you think that your current work leads to morbidity and even SA or if there are limited options to adapt the work to the reduced work capacity.^37^ Those who changed to the care and education sector had slightly higher risk of SA/DP than those who stayed in the trade and retail sector. This is in line with results from other studies of differences in SA/DP by sector, which found that those in the care and education sector have higher rates of SA/DP.^12^ However, the causes of these differences are still unknown. There are also health selection effects into or out of certain occupations based on morbidity.^38,39^ Most of those who were white-collar workers in the trade and retail industry in 2012 were also in the trade and retail industry in 2016 (71%), and 82% were still in the private sector. However, almost half had changed major occupational group. It is thus more common to change occupation than to change branch of industry or sector. More knowledge is needed on how these changes are related to previous and future morbidity.

Our analyses did not include information of actual morbidity. Most people with different diagnoses do not have reduced work capacity to such an extent that they need SA or DP.^40,41^ There is very little research that has investigated this, but one study from Canada based on data from the 1990s suggested that mental disorders were associated with less SA than somatic disorders.^42^ However, the extent to which conclusions from that study can be applied to the situation in Sweden at the present time needs to be investigated.

### Strengths and Limitations

The main strength of this study is the large and population-based cohort including all 192,077 individuals who lived in Sweden all of 2012, were 18 to 67 years old, and were employed in a white-collar occupation by a private company in the trade and retail industries. This means that the study is not based on a sample and that the study population was large enough for subgroup analysis. Another important strength is that the microdata from 3 nationwide administrative registers are of good quality,^43^ meaning that there were no dropouts (all could be followed up from inclusion to emigration, death, or end of follow-up) and that no self-reports, possibly affected by recall bias, were used. Sickness absence/DP diagnoses were determined by the treating physician.

Limitations are the exploratory nature of the study, meaning that we are unable to draw any causal inferences from the research. That we only used SA spells >14 days can be seen as both a strength and a limitation. We also found that many of our included factors had only a weak association with SA/DP. This indicates that there are additional factors of importance that we have not included in this study.

## CONCLUSIONS

In this first exploratory study of SA/DP among white-collar workers in the trade and retail industry, SA was a risk factor for subsequent SA/DP. Mental diagnoses were the leading cause of SA/DP, and the annual number of SA/DP days due to mental diagnoses increased more than those due to other diagnoses. This highlights the importance of studying mental disorders and SA/DP due to mental diagnoses further in this occupational group.

## Supplementary Material

SUPPLEMENTARY MATERIAL
