# Supplementary material for: Sickness Absence and Disability Pension in the Trade and Retail Industry: A Prospective Cohort Study of 192,000 White-Collar Workers in Sweden
Source: J Occup Environ Med. 2022 Jul 29;64(11):912–9. doi: 10.1097/JOM.0000000000002634 (PMC9640291; doi:10.1097/JOM.0000000000002634)
Supplement: SUPPLEMENTARY MATERIAL [file joem-64-912-s001.docx]

**Sickness absence and disability pension in the trade and retail industry: a prospective cohort study of 192,000 white-collar workers in Sweden**

## Abstract

**Objective**: investigate future sickness absence (SA) and disability pension (DP) among privately employed white-collar employees in the trade and retail industry.

**Methods:** A prospective population-based cohort study of all 192,077 such workers in Sweden in 2012, using linked microdata from nationwide registers. Descriptive statistics of annual SA/DP during 2010-2016 and logistic regression for SA/DP in 2016 were used.

**Results:** Women had more mean SA/DP net days/person, however, there were no gender differences in the mean number of net days/person with SA/DP. The mean number of net days/person increased, especially with mental diagnoses. SA in 2012 was the strongest factor associated with SA/DP in 2016 (OR women 3.28, 95% CI 3.09-3.47; men 4.10, 95% CI 3.76-4.48). Work-related factors were weakly associated with future SA/DP.

**Conclusion:** The number of SA/DP net days per person increased, especially SA/DP days due to mental diagnoses.

Key words (7/5-7): Sick leave, sickness absence, disability pension, register study, occupational epidemiology, longitudinal follow-up, mental diagnoses

## Background

Sickness absence (SA) and disability pension (DP) have consequences for society, for insurance agencies, for employers, and for the individual, in terms of financial losses, productivity losses, and a higher risk for future SA/DP (1, 2).

There is very little research about SA/DP among white-collar workers in the trade and retail industry, even though the trade and retail industry employs about 10% of those in activity (i.e., employed, self-employed, or studying) in Sweden (3). Aside from one study on all privately employed white-collar workers in Sweden (4), most research on white-collar workers have utilised the Whitehall-II study of civil servants, of which the majority are white-collar workers (5-7) and the Helsinki Health Study of municipal workers, which includes both white-collar and blue-collar workers (8, 9), both of which only include public employees. These studies have found differences in SA by sociodemographic and socioeconomic factors among white-collar workers (6, 10). The aforementioned study on privately employed white-collar workers in Sweden also found differences by sociodemographic factors and branch of industry (4).

Very little is known about sickness absence in the trade and retail industry, despite its size (11). The trade and retail industry in general has relatively low rates of SA compared to other branches of industry (12). However, white-collar workers in the trade and retail industry comprise a range of different jobs, from executives and managers, to call-centre operatives and administrative staff, and it is quite likely that the average numbers hide a large heterogeneity.

Some previous studies have found that SA rates are generally lower among white-collar workers than blue-collar workers (10, 13). While previous research has to a large extent focused on occupational groups with high SA rates, occupational groups with lower rates make up a substantial part of the labour market, and their SA has great implications for their companies, society, and themselves (4). In many countries, women have higher SA rates than men (14). Studies on the entire population have found differences in SA/DP by job-related factors such as job demands/job control (15-21) and the size of the workplace (22). However, the extent to which this is also the case for privately employed white-collar workers is still unknown.

Currently over 100 different measures of SA are used in the literature (23-25). These mirror the challenges of SA research, such as skewed distributions of both the incidence and duration of SA, that many people have recurring events, that SA spells can be of different durations and grade, that both incidence and duration matters, etc. The different measures use both different numerators (spells, days, individuals, etc.) and different denominators (individuals at work, insured individuals, total individuals in the population etc.). Different measures will lead to different results in the same data, e.g., regarding gender differences in SA (4, 26). Therefore, it is important to use several measures in studies of SA/DP.

**The aim** was to investigate future sickness absence (SA) and/or disability pension (DP) in a cohort of white-collar employees in the trade and retail industry.

**Methods**

This is a population-based prospective cohort study of SA/DP in 2010-2016 among the white-collar workers who in 2012 were aged 18-67 and privately employed in the trade and retail industry, using different measures of their SA/DP.

### Data and study population

We used microdata from three nationwide Swedish administrative registers, linked at individual level by the use of the Personal Identity Number (PIN, a unique ten-digit number assigned to all residents in Sweden): (1) Longitudinal Integration Database for Health Insurance and Labour Market Studies (LISA) held by Statistics Sweden; (2) MicroData for Analysis of the Social Insurance database (MiDAS) held by the Social Insurance Agency; and (3) the Cause of Death Register held by the National Board of Health and Welfare.

The study population was all who were aged 18-67 years and registered as living in Sweden in both 2011 and 2012, had an occupational code according to the Swedish Standard for Occupational Classification (SSYK) that indicated a white-collar occupation, were employed at a private sector company in the trade and retail industry according to the Swedish Standard Industrial Classification (SNI), and during 2012 had income from work, parental benefits, and/or SA/DP that amounted to at least 7920 SEK (75% of the necessary income level to qualify for SA benefits from the Social Insurance Agency). The limit of 75% of the minimum income to qualify for SA benefits was set since in many cases SA benefits cover about 75% of the work income; without this adjustment, people with low incomes and long-term SA might have fallen below the minimum income level to be included in the study (27). Those who had full-time DP all of 2012 were excluded, while those who had SA or partial DP were included. This gave a cohort of 192,077 individuals.

In all analyses, individuals were excluded from the year after they emigrated or died, and they were also excluded from the analyses for a year if they did not have income from work, parental leave benefits or SA/DP that year that exceeded 75% of the minimum income needed to qualify for SA benefits.

*Variables*

We used information on the following variables from 2012 unless otherwise noted: *Sex*: woman or man; *Age:* 18-24, 25-34, 35-44, 45-54, 55-64, or 65-67 years; *Country of birth:* Sweden, other Nordic country, other EU25, or rest of world including missing; *Educational level:* compulsory school (≤9 years or missing), high school (10-12 years), or college/university (≥13 years); *Family situation:* married/cohabiting with children at home, married cohabiting without children at home, single with children at home, or single without children at home; *Type of living area:* large city (Stockholm, Gothenburg, or Malmö), medium-sized town >90,000 inhabitants within 30 km of city centre), or small town/rural (<90,000 inhabitants within 30 km of city centre).

For information on *job demands/job control*, we used a psychosocial job exposure matrix (JEM) (28) (for more details, see (29)). We categorised individuals into nine groups: high demands/high control, high demands/medium control, high demands/low control, medium demands/high control, medium demands/medium control, medium demands/low control, low demands/high control, low demands/medium control, and low demands/low control. *Workplace size* was categorised into 1-9 employees, 10-49 employees, 50-99 employees, 100-499 employees and ≥500 employees.

*Branch of industry* in 2016 based on SNI was categorised as: trade and retail, manufacturing, services, transport, construction and installation, care and education, or restaurants and hotels. *Change of occupation* between 2012 and 2016 based on SSYK was categorised as: Change within occupational category or no change, Change of occupational category within the same major occupational group, Change to a higher major occupational group (e.g., from 2 to 1), Change to a lower major occupational group (e.g., from 1 to 2). *Occupational sector* in 2016 was categorised into municipal, region, state, private, or other.

Diagnosis-specific SA/DP was categorised into the following *Diagnosis groups:* mental diagnoses (ICD-10 codes F00-F99, and Z73), musculoskeletal diagnoses (M00-M99), injuries (S00-T98 and V01-Y98), cancer (C00-D48), cardiovascular diagnoses (I00-I99), pregnancy-related diagnoses (among women: O00-O99 and N96), or other diagnoses (all other diagnoses, including missing).

*Measures*

We calculated the following SA/DP measures:

- annual numbers and prevalence of people with SA/DP in 2010-2016
- annual mean number of SA/DP net days per person in 2010-2016
- annual mean number of SA/DP net days per person with SA/DP in 2010-2016
- annual mean number of SA/DP net days in different diagnosis groups in 2010-2016
- odds ratios for having SA/DP in 2016.

We also ran sensitivity analyses for the odds ratios of having SA/DP in 2016, excluding all those who had any SA or DP in 2012.

The project was approved by the Regional Ethical Review Board of Stockholm, Sweden.

*Public sickness absence insurance in Sweden*

All people living in Sweden aged ≥16 years with an income from work or unemployment benefits are covered by the national public SA insurance, and can claim SA benefits for a reduced work capacity due to disease or injury, without an upper age limit, although some restrictions to the length of SA apply after age 65. After a first qualifying day, the employer provides sick pay for days 2-14 of the SA spell, after which SA benefits are paid by the Social Insurance Agency. Self-employed have more qualifying days. Unemployed get SA benefits from the Social Insurance Agency after the first qualifying day. A physician certificate is required after 7 days of self-certification. In this study, data on SA with benefits from the Social Insurance Agency were used. SA spells ≤14 days were not included in the study, so as not to introduce bias regarding those who might have been unemployed. SA spells could be ongoing for years. All residents in Sweden aged 19-64 years, whose work capacity is permanently or long-term reduced due to disease or injury, can be granted DP from the Social Insurance Agency. SA benefits cover 80% and DP benefits 64% of lost income, both up to a certain level.

Both SA and DP can be granted for part- or full-time (25, 50, 75, or 100% of ordinary work hours), this means that people can be on partial SA and DP at the same time. Therefore, we used net days so that partial days of SA/DP were combined. The number of net days were calculated using the number of gross days with benefits multiplied by the extent of absence (i.e., 25, 50, 75, or 100%), e.g., two days of absence for 75% were counted as 1.5 net days.

## Results

Table 1 shows the sociodemographic characteristics of the cohort in 2012. There was a slightly higher proportion of men (55.56%) and the vast majority were aged 25-64.

The majority among both women and men lived in large cities (Stockholm, Gothenburg, or Malmö), and were born in Sweden. A very small proportion (5.60% of women and 9.21% of men) had only elementary education, while 44.89% of women and 37.10% of men had at least some college/university education. The majority were married/living with partner with children <18 years living at home, but more than twice the proportion of women (9.40%) than men (3.72%) were single with children living at home.

Supplementary Figure 1, <http://links.lww.com/JOM/B149>, shows the distribution of job demands/control for all and for women and men in a kernel density plot. Among the entire population, the distribution seemed fairly even, however, while the level of demands was equally distributed among women and men, there were far more women in jobs with low control, and far more men in jobs with high control.

Figure 1 shows the proportions of women and men who had at least some SA/DP during each of the study years 2010-2016. Each year, the proportions who had SA/DP were higher among women (8-13%, depending on year) than men (3-6% depending on year). The proportion of the cohort who had SA/DP increased slightly between 2010 (8% of women and 4% of men) and 2016 (15% of women and 6% of men).

Figure 2 shows the annual mean number of SA/DP days per person (A) as well as per person with SA/DP the respective year (B) during 2010-2016. The mean number of SA/DP days increased each year, from 6.6 mean days among women in 2010 to 13.5 in 2016, and from 2.7 to 5.7 among men, except for a slight dip in 2014 among both women and men. Women had more SA/DP days than men each year, and the increase in SA/DP days was slightly larger among women than men.

The annual mean number of SA/DP days per person with SA/DP was, as expected, much higher than the number of net days per employed person, but also increased from 80.7 mean days among women in 2012 to 100.3 in 2016, and from 73.9 to 96.0 days among men, with a slight dip in 2014. There were larger sex differences in the mean number of SA/DP days per employed person, than the mean number of SA/DP days per person with SA/DP.

In Figure 3, the annual mean number of SA/DP days is presented by diagnosis groups. The mean number of days increased over the studied years among both women and men, especially days due to mental diagnoses. The increase in the other diagnosis groups was smaller, leading to mental diagnoses constituting an increasing proportion of SA/DP days.

Table 2 presents OR and 95% CIs for the risk of having SA/DP in 2016. Men had a lower risk than women of such SA/DP (OR 0.46; 95% CI 0.44-0.49). Those who were aged 65-67 years in 2012 were much less likely to have SA in 2016 (OR 0.09; 95% CI 0.05-0.18) than those aged 35-44. Otherwise the ORs by age were relatively close to 1, rarely over 1.50 or under 0.67, with one exception: OR for men aged 55-64 was 1.80 (95% CI 1.64-1.97).

Those with only elementary education had a higher risk of SA/DP than those with at least some university/college education, and this was stronger among men (OR 1.83; 95% CI 1.65-2.02) than among women (OR 1.54, 05% CI 1.40-1.69).

Having had SA in 2012 was associated with a much higher risk of having SA/DP in 2016, and again this was stronger among men (OR 4.10; 95% CI 3.76-4.48) than among women (OR 3.28; 95% CI 3.09-3.47).

There were no large differences in the risk of SA/DP by the job-related factors in 2012 (size of company, job demands/control), nor by the job-related factors in 2016 (change of occupation, branch of industry, or sector); ORs were between 1.50 and 0.67. For men, there was a small but significant association between low control and a higher risk of SA/DP (ORs range 1.10-1.23), whereas high control was associated with a lower risk (ORs range 0.85-0.90), for all levels of job demands. However, for women there were no such significant associations with job demands/control. Just over half the population did not change occupation or changed within the major occupational group (50.3%), 71% were still in the trade and retail industry, and 83% were still in the private sector at the end of follow-up.

Excluding those who had any SA/DP in 2012 did not change the magnitude of the estimates for the other variables in any major way (Supplementary Table 1, <http://links.lww.com/JOM/B150>).

## Discussion

In this first exploratory prospective cohort study of SA/DP among all privately employed white-collar workers in the trade and retail industry in Sweden, we found that the rate of people with SA/DP was low and relatively stable over the years, while the mean number of SA/DP days increased slightly. The mean number of days per person with SA/DP increased especially, indicating that it was rather duration of SA/DP that increased, than numbers of people on SA/DP. We also found that the mean number of SA/DP days due to mental diagnoses increased more than the mean number of such days due to other diagnoses. While the women had slightly more SA/DP days/year than men in the entire cohort, there were no such gender differences in the mean number of SA/DP days among those who had SA/DP. That is, even though more women had SA/DP than men, there were no sex differences in length of SA/DP among those who had SA/DP.

Mental diagnoses were the most common SA/DP diagnoses, when somatic diagnoses were categorized in smaller groups. This has been found in several other studies of white-collar workers, although they have been either general studies among white-collar workers (4) or of public employees, not specifically studied among people employed in the private trade and retail industry (30). SA spells due to mental diagnoses often become longer than SA spells due to other diagnoses in countries where long SA spells are possible, such as Sweden (31, 32). Therefore, it is of interest to further study SA due to mental diagnoses in this occupational group. Which mental diagnoses are the most common, and which have the greatest risk of SA spells becoming long? Which interventions can prevent that such SA spells become long-term? To what extent do people on SA due to mental diagnoses later have SA due to other diagnoses, and vice versa? How does the prevalence of SA due to mental diagnoses among white-collar workers in the trade and retail industry compare to white-collar workers in other branches of industry?

The mean number of SA/DP days per person with SA/DP increased more than both the proportion of individuals with SA/DP and slightly more than the mean number of net days of SA/DP per employed person. This indicates that the length or extent of SA/DP increased for those who were on SA/DP. The increase in SA/DP length could be related to the cohort getting older, as older age is associated with longer SA spells (33), and also to that SA spells due to mental disorders tend to be longer than spells due to other diagnoses (31, 32). Compared to white-collar workers on the entire labour market, those in the trade and retail industry had slightly fewer SA/DP days, both among all and per person with SA/DP (34). That women have far more SA/DP days per person in total than men do, but a similar number of days per person with SA/DP, is in line with previous results on white-collar workers on the entire labour market (4, 34, 35).

The strongest factors that predicted SA/DP in 2016 were SA in 2012, low educational level, and female sex. We found no strong associations between the included job-related factors (job demands/control, workplace size, change of occupation, branch of industry in 2016 or sector in 2016) and SA/DP in 2016. This indicates that there are other factors, either job-related, sociodemographic, socioeconomic, or morbidity-related, that explains the differences in SA/DP in this group, and this should be studied further. That job demands/control was not strongly associated with future SA/DP could possibly be due to the distribution of demands/control, which was much more even than in the entire population (29). We also found a much clearer differentiation by sex than previous studies have found in the general population, especially regarding the level of job control, where women were predominantly located in occupations with low control and men in occupations with high control. This is not as clear in studies of the entire population (29, 36). The sex differences in the level of control could also possibly contribute to the sex differences in SA/DP, however, this needs to be investigated further. That change of occupation, branch of industry, or sector in 2016 were not strongly associated with SA/DP could be related to that most people were still in the same occupation, in the trade and retail industry and private sector. Changing your job can be a part of work rehabilitation, especially if you think that your current work leads to morbidity and even SA, or if there are limited options to adapt the work to the reduced work capacity (37). Those who changed to the care and education sector had slightly higher risk of SA/DP than those who stayed in the trade and retail sector. This is in line with results from other studies of differences in SA/DP by sector, which found that those in the care and education sector have higher rates of SA/DP (12). However, the causes of these differences are still unknown. There are also health selection effects into or out of certain occupations based on morbidity (38, 39). Most of those who were white-collar workers in the trade and retail industry in 2012 were also in the trade and retail industry in 2016 (71%), and 82% were still in the private sector. However, almost half had changed major occupational group. It is thus more common to change occupation than to change branch of industry or sector. More knowledge is needed on how these changes are related to previous and future morbidity.

Our analyses did not include information of actual morbidity. Most people with different diagnoses do not have reduced work capacity to such an extent that they need SA or DP (40, 41). There is very little research that has investigated this, but one study from Canada based on data from the 1990s suggested that mental disorders were associated with less SA than somatic disorders (42). However, the extent to which conclusions from that study can be applied to the situation in Sweden at the present time needs to be investigated.

*Strengths and limitations*

The main strength of this study is the large and population-based cohort including all 192,077 individuals who lived in Sweden all of 2012, were 18-67 years old, and were employed in a white-collar occupation by a private company in the trade and retail industries. This means that the study is not based on a sample, and that the study population was large enough for subgroup analysis. Another important strength is that microdata from three nationwide administrative registers of good quality (43), meaning that there were no drop-outs (all could be followed up from inclusion to emigration, death or end of follow-up) and that no self-reports, possibly affected by recall bias, were used. SA/DP diagnoses were determined by the treating physician.

Limitations are the exploratory nature of the study, meaning that we are unable to draw any causal inferences from the research. That we only used SA spells >14 days can be seen as both a strength and a limitation. We also found that many of our included factors had only a weak association with SA/DP. This indicates that there are additional factors of importance that we have not included in this study.

## Conclusion

In this first exploratory study of SA/DP among white-collar workers in the trade and retail industry, SA was a risk factor for subsequent SA/DP. Mental diagnoses were the leading cause of SA/DP and the annual number of SA/DP days due to mental diagnoses increased more than those due to other diagnoses. This highlights the importance of studying mental disorders and SA/DP due to mental diagnoses further in this occupational group.

## References

1. Alexanderson K, Norlund A. Swedish Council on Technology Assessment in Health Care (SBU). Chapter 1. Aim, background, key concepts, regulations, and current statistics. Scand J Public Health. 2004;32(63 suppl):12-30.

2. Walker R. Social security and welfare. Concepts and comparisons. In: Gladstone D, editor. Introducing Social Policy. Milton Keynes: Open University Press; 2011.

3. Svensk Handel. Läget i handeln: 2021 års rapport om branschens ekonomiska utveckling. Stockholm: Svensk Handel; 2021.

4. Farrants K, Alexanderson K. Sickness absence among privately employed white-collar workers: a total population study in Sweden. Scand J Publ Health. 2021;49(2):159-67.

5. Head J, Ferrie JE, Alexanderson K, Westerlund H, Vahtera J, Kivimaki M. Diagnosis-specific sickness absence as a predictor of mortality: the Whitehall II prospective cohort study. British Medical Journal. 2008;337.

6. Feeney A, North F, Head J, Canner R, Marmot M. Socioeconomic and sex differentials in reason for sickness absence from the Whitehall II Study. Occup Environ Med. 1998;55(2):91-8.

7. Hemingway H, Shipley MJ, Stansfeld S, Marmot M. Sickness absence from back pain, psychosocial work characteristics and employment grade among office workers. Scandinavian Journal of Work Environment & Health. 1997;23(2):121-9.

8. Laaksonen M, Martikainen P, Rahkonen O, Lahelma E. Explanations for gender differences in sickness absence: evidence from middle-aged municipal employees from Finland. Occupational and Environmental Medicine. 2008;65(5):325-30.

9. Leinonen T, Pietilainen O, Laaksonen M, Rahkonen O, Lahelma E, Martikainen P. Occupational social class and disability retirement among municipal employees - the contribution of health behaviors and working conditions. Scandinavian Journal of Work Environment & Health. 2011;37(6):464-72.

10. Piha K, Laaksonen M, Martikainen P, Rahkonen O, Lahelma E. Interrelationships between education, occupational class, income and sickness absence. Eur J Public Health. 2010;20(3):276-80.

11. Farrants K, Alexanderson K. Sjukskrivning och sjuk- och aktivitetsersättning över fem år i en grupp tjänstemän i handeln. Stockholm: Handelsrådet; 2022.

12. Sjukfrånvaro per bransch och sektor: Statistikbilaga till pressmeddelande, november 2016 [Sickness absence per branch of industry and sector: Statistical appendix to press release, November 2016]. In Swedish: Social Insurance Agency; 2016.

13. Väänänen A, Kalimo R, Toppinen-Tanner S, Mutanen P, Peiró JM, Kivimäki M, Vahtera J. Role clarity, fairness, and organizational climate as predictors of sickness absence: A prospective study in the private sector. Scandinavian Journal of Public Health. 2004;32(6):426-34.

14. Allebeck P, Mastekaasa A. Swedish Council on Technology Assessment in Health Care (SBU). Chapter 5. Risk factors for sick leave - general studies. Scand J Publ Health. 2004;32:49-108.

15. de Vries H, Fishta A, Weikert B, Rodriguez Sanchez A, Wegewitz U. Determinants of Sickness Absence and Return to Work Among Employees with Common Mental Disorders: A Scoping Review. J Occup Rehabil. 2018;28(3):393-417.

16. Williams-Whitt K, White MI, Wagner SL, Schultz IZ, Koehn C, Dionne CE, Koehoorn M, Harder H, Pasca R, Warje O, Hsu V, McGuire L, Schulz W, Kube D, Hook A, Wright MD. Job demand and control interventions: a stakeholder-centered best-evidence synthesis of systematic reviews on workplace disability. Int J Occup Environ Med. 2015;6(2):61-78.

17. Knardahl S, Johannessen HA, Sterud T, Harma M, Rugulies R, Seitsamo J, Borg V. The contribution from psychological, social, and organizational work factors to risk of disability retirement: a systematic review with meta-analyses. BMC public health. 2017;17(1):176.

18. Bourbonnais R, Brisson C, Moisan J, Vézina M. Job strain and psychological distress in white collar workers. Scand J Work Env Health. 1996(2):139-45.

19. Robroek SJ, Schuring M, Croezen S, Stattin M, Burdorf A. Poor health, unhealthy behaviors, and unfavorable work characteristics influence pathways of exit from paid employment among older workers in Europe: a four year follow-up study. Scand J Work Environ Health. 2013;39(2):125-33.

20. Mortensen J, Dich N, Lange T, Alexanderson K, Goldberg M, Head J, Kivimaki M, Madsen IEH, Rugulies R, Vahtera J, Zins M, Rod NH. Job strain and informal caregiving as predictors of long-term sickness absence: A longitudinal multi-cohort study. Scandinavian Journal of Work Environment & Health. 2017;43(1):5-14.

21. Samuelsson A, Ropponen A, Alexanderson K, Svedberg P. Psychosocial working conditions, occupational groups, and risk of disability pension due to mental diagnoses: a cohort study of 43 000 Swedish twins. Scandinavian Journal of Work Environment & Health. 2013;39(4):351-60.

22. Barmby T, Stephen G. Worker Absenteeism: Why Firm Size May Matter. The Manchester School. 2000;68(5):568-77.

23. Hensing G, Alexanderson K, Allebeck P, Bjurulf P. How to measure sickness absence? Literature review and suggestion of five basic measures. Scandinavian Journal of Social Medicine. 1998;26(2):133-44.

24. Alexanderson K, Hensing G. More and better research needed on sickness absence. Scand J Public Health. 2004;32(5):321-3.

25. Hensing G. The measurements of sickness absence – a theoretical perspective. Norsk Epidemiologi. 2009;19(2):147-51.

26. Hensing G, Alexanderson K, Allebeck P, Bjurulf P. Sick-leave due to psychiatric disorder: higher incidence among women and longer duration for men. Br J Psychiatry. 1996;169(6):740-6.

27. Farrants K, Marklund S, Kjeldgård L, Head J, Alexanderson K. Sick leave before and after the age of 65 among those in paid work in Sweden in 2000 and 2005: a register-based cohort study. Int J Med Res. 2017;46(2):564-77.

28. Fredlund P, Hallqvist J, Diderichsen F. Psychosocial job exposure matrix. An updated version of a classification system for work-related psychosocial exposure. Stockholm: Swedish National Institute for Working Life; 2000.

29. Norberg J, Alexanderson K, Framke E, Rugulies R, Farrants K. Job demands and control and sickness absence, disability pension, and unemployment among 2,194,692 individuals in Sweden. Scand J Publ Health. 2019;48(2):125-33.

30. Leinonen T, Viikari-Juntura E, Husgafvel-Pursiainen K, Solovieva S. Cause-specific sickness absence trends by occupational class and industrial sector in the context of recent labour market changes: a Finnish panel data study. BMJ Open. 2018;8(4):e019822.

31. Lidwall U. Sick leave diagnoses and return to work: a Swedish register study. Disabil Rehabil. 2015;37(5):396-410.

32. Hensing G, Spak F. Psychiatric disorders as a factor in sick-leave due to other diagnoses. A general population-based study. The British Journal of Psychiatry. 1998;172(3):250-6.

33. Beemsterboer W, Stewart R, Groothoff J, Nijhuis F. A literature review on sick leave determinants (1984-2004). Int J Occup Med Environ Health. 2009;22(2):169-79.

34. Farrants K, Alexanderson K. Sjukfrånvaro bland privatanställda tjänstemän 2012-2018. Stockholm: Karolinska Institutet; 2022.

35. Farrants K, Sondén A, Nilsson K, Alexanderson K. Sjukfrånvaro bland privatanställda tjänstemän. Stockholm: Karolinska Institutet; 2018.

36. Salonen L, Alexanderson K, Rugulies R, Framke E, Niemelä M, Farrants K. Combinations of Job Demands and Job Control and Future Trajectories of Sickness Absence and Disability Pension An 11-year Follow-up of Two Million Employees in Sweden. J Occup Environ Med. 2020;62(10):795-802.

37. Nordström K, Ekberg K, Hemmingsson T, Johansson G. Sick leave and the impact of job-to-job mobility on the likelihood of remaining on the labour market - a longitudinal Swedish register study. BMC public health. 2014;14(1):305.

38. Li C-Y, Sung F-C. A review of the healthy worker effect in occupational epidemiology. Occupational Medicine. 1999;49(4):225-9.

39. Bambra C. Work, Worklessness, and the Political Economy of Health. Oxford: Oxford University Press; 2011.

40. Wikman A, Marklund S, Alexanderson K. Illness, disease, and sickness absence: an empirical test of differences between concepts of ill health. Journal of epidemiology and community health. 2005;59(6):450-4.

41. Björkenstam C, Alexanderson K, Wiberg M, Hillert J, Tinghög P. Heterogeneity of sickness absence and disability pension trajectories among individuals with MS. Multiple sclerosis journal - experimental, translational and clinical. 2015;1:2055217315595638.

42. Dewa CS, Lin E. Chronic physical illness, psychiatric disorder and disability in the workplace. Soc Sci Med. 2000;51(1):41-50.

43. Ludvigsson JF, Almqvist C, Bonamy AK, Ljung R, Michaelsson K, Neovius M, Stephansson O, Ye W. Registers of the Swedish total population and their use in medical research. Eur J Epidemiol. 2016;31(2):125-36.

**Figure legends:**

**Figure 1: Proportions of white-collar workers in the trade and retail industry in the 2012 cohort who had any SA/DP, in each of the years 2010-2016, stratified by sex**

**Figure 2: Mean number of sickness absence (SA) and disability pension (DP) days per employed person and year (A), and per person with SA/DP in the respective year (B)**

**Figure 3: Mean number of net days with sickness absence (SA) and disability pension (DP), respectively, per person and year in different diagnosis groups**
