# Supplementary material for: Sickness Absence and Disability Pension in the Trade and Retail Industry: A Prospective Cohort Study of 192,000 White-Collar Workers in Sweden
Source: J Occup Environ Med. 2022 Jul 29;64(11):912–9. doi: 10.1097/JOM.0000000000002634 (PMC9640291; doi:10.1097/JOM.0000000000002634)
Supplement: SUPPLEMENTARY MATERIAL [file joem-64-912-s002.docx]

**Supplementary Table 1: Crude and mutually adjusted odds ratios (OR) and 95% confidence intervals (CI) over the association between sociodemographic and job-related factors in 2012 with sickness absence (SA) and/or disability pension (DP) in 2016, when excluding those with any SA or DP in 2012, among all and stratified by sex.**

|  | **All** | | **Women** | | **Men** | |
| --- | --- | --- | --- | --- | --- | --- |
|  | **Crude OR (95% CI)** | **Adjusted OR (95% CI)** | **Crude OR (95% CI)** | **Adjusted OR (95% CI)** | **Crude OR (95% CI)** | **Adjusted OR (95% CI)** |
| **All** | **192,077** |  | **85,356** |  | **106,721** |  |
| *Sex* |  |  |  |  |  |  |
| Women | Ref | Ref |  |  |  |  |
| Men | 0.44 (0.43-0.46) | 0.45 (0.43-0.47) |  |  |  |  |
| *Age* |  |  |  |  |  |  |
| 18-24 years | 1.06 (0.97-1.16) | 0.90 (0.81-1.00) | 0.94 (0.84-1.05) | 0.89 (0.79-1.02) | 0.98 (0.82-1.16) | 0.85 (0.70-1.03) |
| 25-34 years | 1.10 (1.05-1.16) | 1.07 (1.01-1.13) | 1.11 (1.05-1.18) | 1.13 (1.06-1.21) | 0.94 (0.86-1.02) | 0.91 (0.83-1.00) |
| 35-44 years | Ref | Ref | Ref | Ref | Ref | Ref |
| 45-54 years | 1.12 (1.07-1.17) | 1.09 (1.04-1.15) | 1.04 (0.98-1.11) | 0.97 (0.91-1.04) | 1.30 (1.21-1.40) | 1.32 (1.22-1.43) |
| 55-64 years | 0.96 (0.91-1.02) | 1.18 (1.1-1.26) | 0.73 (0.67-0.79) | 0.85 (0.77-0.94) | 1.40 (1.28-1.52) | 1.71 (1.54-1.89) |
| 65-67 years | 0.06 (0.03-0.11) | 0.12 (0.06-0.24) | 0.05 (0.02-0.12) | 0.09 (0.03-0.25) | 0.08 (0.03-0.18) | 0.17 (0.06-0.45) |
| *Type of living area* |  |  |  |  |  |  |
| Large city (Stockholm, Gothenburg, Malmö) | Ref | Ref | Ref | Ref | Ref | Ref |
| Medium-sized town (>90,000 inhabitants) | 1.04 (1.00-1.08) | 1.03 (0.99-1.08) | 1.03 (0.98-1.09) | 1.00 (0.95-1.06) | 1.16 (1.09-1.24) | 1.07 (1.00-1.15) |
| Rural (<90,000 inhabitants) | 1.12 (1.07-1.18) | 1.09 (1.03-1.15) | 1.10 (1.03-1.17) | 1.07 (1.00-1.15) | 1.25 (1.16-1.36) | 1.11 (1.02-1.21) |
| *Educational level* |  |  |  |  |  |  |
| Elementary (≤9 years) | 1.38 (1.29-1.48) | 1.64 (1.52-1.77) | 1.33 (1.21-1.48) | 1.53 (1.37-1.71) | 2.01 (1.82-2.22) | 1.85 (1.66-2.05) |
| High school (10-12 years) | 1.24 (1.19-1.29) | 1.30 (1.25-1.36) | 1.2 (1.15-1.26) | 1.24 (1.17-1.31) | 1.57 (1.47-1.67) | 1.51 (1.40-1.62) |
| University/college (>12 years) | Ref | Ref | Ref | Ref | Ref | Ref |
| *Birth country* |  |  |  |  |  |  |
| Sweden | Ref | Ref | Ref | Ref | Ref | Ref |
| Other Nordic countries | 1.19 (1.06-1.34) | 1.08 (0.95-1.23) | 1.05 (0.91-1.22) | 1.09 (0.94-1.28) | 1.17 (0.96-1.44) | 1.09 (0.88-1.36) |
| Other EU25 | 0.98 (0.85-1.12) | 0.99 (0.85-1.15) | 0.96 (0.81-1.14) | 1.03 (0.86-1.23) | 0.88 (0.69-1.12) | 0.94 (0.72-1.22) |
| Rest of the world | 1.29 (1.19-1.39) | 1.27 (1.17-1.38) | 1.21 (1.10-1.34) | 1.22 (1.10-1.35) | 1.18 (1.03-1.36) | 1.35 (1.17-1.57) |
| *Family situation* |  |  |  |  |  |  |
| Married/cohabitant without children at home | Ref | Ref | Ref | Ref | Ref | Ref |
| Married/cohabitant with children at home | 1.04 (0.98-1.10) | 0.92 (0.86-0.99) | 1.13 (1.04-1.22) | 0.86 (0.78-0.94) | 0.97 (0.88-1.06) | 1.00 (0.90-1.11) |
| Single without children at home | 1.16 (1.09-1.24) | 1.06 (0.98-1.13) | 1.24 (1.14-1.34) | 1.01 (0.92-1.11) | 1.07 (0.98-1.18) | 1.13 (1.01-1.26) |
| Single with children at home | 1.82 (1.68-1.97) | 1.26 (1.15-1.38) | 1.66 (1.51-1.83) | 1.22 (1.10-1.36) | 1.37 (1.18-1.60) | 1.27 (1.08-1.50) |
| *Number of employees at workplace* |  |  |  |  |  |  |
| 1-9 employees | 1.11 (1.06-1.16) | 1 (0.95-1.05) | 1.06 (1.00-1.13) | 1.01 (0.95-1.08) | 1.05 (0.98-1.13) | 0.98 (0.91-1.05) |
| 10-49 employees | Ref | Ref | Ref | Ref | Ref | Ref |
| 50-99 employees | 0.96 (0.91-1.02) | 0.98 (0.92-1.04) | 0.92 (0.85-1.00) | 0.94 (0.86-1.02) | 0.98 (0.90-1.08) | 1.03 (0.94-1.14) |
| 100-499 employees | 0.98 (0.92-1.03) | 0.97 (0.92-1.03) | 0.96 (0.90-1.02) | 0.99 (0.92-1.06) | 0.84 (0.77-0.92) | 0.94 (0.86-1.03) |
| ≥500 employees | 1.05 (0.96-1.14) | 1.08 (0.99-1.19) | 1.05 (0.94-1.17) | 1.10 (0.98-1.23) | 0.82 (0.70-0.96) | 1.01 (0.85-1.19) |
| *Control/demands* |  |  |  |  |  |  |
| Low control, low demands | 1.44 (1.34-1.55) | 1.01 (0.94-1.10) | 0.99 (0.90-1.10) | 0.97 (0.88-1.08) | 1.06 (0.91-1.22) | 1.11 (0.95-1.30) |
| Low control, medium demands | 1.37 (1.26-1.48) | 1.02 (0.93-1.11) | 0.99 (0.89-1.10) | 0.96 (0.86-1.07) | 1.14 (1.00-1.31) | 1.21 (1.04-1.40) |
| Low control, high demands | 1.56 (1.45-1.67) | 1.03 (0.95-1.11) | 1.00 (0.91-1.10) | 0.98 (0.89-1.09) | 1.21 (1.02-1.43) | 1.26 (1.06-1.51) |
| Medium control, Low demands | 1.08 (1.00-1.17) | 0.98 (0.90-1.07) | 0.94 (0.84-1.05) | 0.94 (0.84-1.06) | 1.00 (0.89-1.12) | 1.02 (0.91-1.15) |
| Medium control, medium demands | Ref | Ref | Ref | Ref | Ref | Ref |
| Medium control, high demands | 1.25 (1.16-1.34) | 1.01 (0.94-1.10) | 1.04 (0.94-1.15) | 1.03 (0.93-1.14) | 0.89 (0.79-1.01) | 0.92 (0.80-1.04) |
| High control, low demands | 0.82 (0.76-0.89) | 0.98 (0.90-1.07) | 1.00 (0.85-1.18) | 1.01 (0.86-1.20) | 0.97 (0.88-1.08) | 0.93 (0.84-1.04) |
| High control, medium demands | 0.74 (0.68-0.80) | 0.93 (0.85-1.01) | 0.79 (0.65-0.97) | 0.80 (0.65-0.99) | 0.94 (0.85-1.03) | 0.91 (0.82-1.01) |
| High control, high demands | 0.83 (0.76-0.90) | 0.92 (0.84-1.01) | 0.94 (0.81-1.09) | 0.92 (0.79-1.07) | 0.91 (0.81-1.02) | 0.89 (0.79-1.00) |
| *Job change* |  |  |  |  |  |  |
| Change within occupational category or no change (n=86,858) | Ref | Ref | Ref | Ref | Ref | Ref |
| Change of occupational category within the same SSYK chapter (n=40,535) | 1.11 (1.06-1.16) | 1.02 (0.97-1.07) | 1.07 (1.01-1.14) | 1.01 (0.95-1.08) | 1.01 (0.94-1.09) | 0.99 (0.92-1.08) |
| Change to a higher SSYK chapter (e.g., from 2 to 1) (n=18,467) | 0.95 (0.89-1.01) | 0.89 (0.83-0.95) | 0.98 (0.91-1.06) | 0.92 (0.85-1.00) | 0.79 (0.71-0.88) | 0.80 (0.71-0.9) |
| Change to a lower SSYK chapter (e.g,. from 1 to 2) (n=29,701) | 1.26 (1.20-1.33) | 1.14 (1.08-1.2) | 1.23 (1.15-1.31) | 1.09 (1.01-1.17) | 1.30 (1.21-1.4) | 1.15 (1.06-1.24) |
| *Branch of industry in 2016* |  |  |  |  |  |  |
| Construction (n=2144) | 0.97 (0.82-1.14) | 1.05 (0.89-1.25) | 0.71 (0.52-0.95) | 0.67 (0.49-0.91) | 1.42 (1.16-1.73) | 1.40 (1.14-1.72) |
| Hospitality (n=808) | 1.34 (1.06-1.70) | 1.22 (0.95-1.55) | 1.08 (0.81-1.44) | 1.06 (0.78-1.43) | 1.53 (1.02-2.28) | 1.64 (1.08-2.47) |
| Manufacturing (n=8704) | 0.86 (0.79-0.94) | 0.97 (0.88-1.06) | 0.90 (0.80-1.02) | 0.96 (0.85-1.09) | 0.93 (0.82-1.05) | 0.99 (0.87-1.13) |
| Unknown (N=9019) | 0.82 (0.75-0.89) | 0.90 (0.68-1.19) | 0.70 (0.62-0.78) | 0.89 (0.62-1.28) | 0.97 (0.85-1.11) | 0.90 (0.57-1.41) |
| Services (n=22,287) | 0.91 (0.86-0.96) | 0.91 (0.86-0.97) | 0.89 (0.83-0.96) | 0.88 (0.82-0.95) | 0.87 (0.80-0.96) | 0.96 (0.87-1.07) |
| Transport (n=1103) | 0.94 (0.75-1.19) | 1.03 (0.81-1.30) | 0.96 (0.69-1.33) | 1.00 (0.72-1.39) | 1.05 (0.75-1.46) | 1.05 (0.75-1.48) |
| Care and education (n=5393) | 1.65 (1.52-1.80) | 1.17 (1.04-1.32) | 1.31 (1.19-1.44) | 1.14 (0.99-1.31) | 1.44 (1.18-1.75) | 1.29 (1.01-1.65) |
| Trade and retail (n=126,103) | 0.97 (0.82-1.14) | 1.05 (0.89-1.25) | 0.71 (0.52-0.95) | 0.67 (0.49-0.91) | 1.42 (1.16-1.73) | 1.40 (1.14-1.72) |
| *Sector in 2016* |  |  |  |  |  |  |
| Municipal (n=3402) | 1.86 (1.68-2.07) | 1.4 (1.22-1.59) | 1.47 (1.31-1.66) | 1.37 (1.18-1.6) | 1.66 (1.33-2.08) | 1.52 (1.17-1.99) |
| Region (n=1005) | 1.84 (1.53-2.23) | 1.37 (1.11-1.70) | 1.42 (1.16-1.75) | 1.34 (1.06-1.70) | 1.48 (0.92-2.40) | 1.45 (0.87-2.43) |
| State (n=5096) | 1.58 (1.45-1.73) | 1.34 (1.22-1.47) | 1.24 (1.12-1.37) | 1.33 (1.20-1.48) | 1.31 (1.05-1.62) | 1.41 (1.13-1.75) |
| Other (n=3168) | 1.26 (1.11-1.42) | 1.17 (1.03-1.33) | 1.12 (0.96-1.31) | 1.13 (0.96-1.33) | 1.29 (1.05-1.58) | 1.24 (1.01-1.53) |
| Private sector (n=148,730) | Ref | Ref | Ref | Ref | Ref | Ref |
