# Supplementary material for: Sickness Absence and Disability Pension in the Trade and Retail Industry: A Prospective Cohort Study of 192,000 White-Collar Workers in Sweden
Source: J Occup Environ Med. 2022 Jul 29;64(11):912–9. doi: 10.1097/JOM.0000000000002634 (PMC9640291; doi:10.1097/JOM.0000000000002634)
Supplement: SUPPLEMENTARY MATERIAL [file joem-64-912-s003.docx]

A)


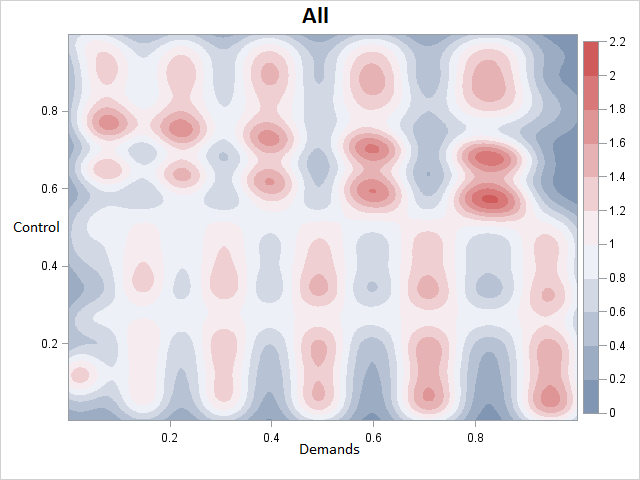


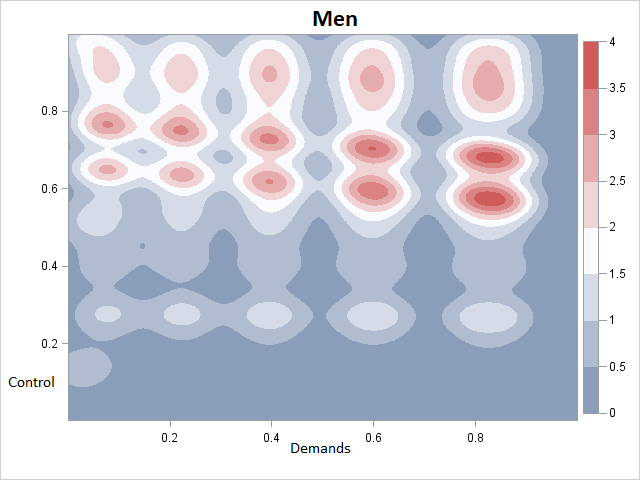

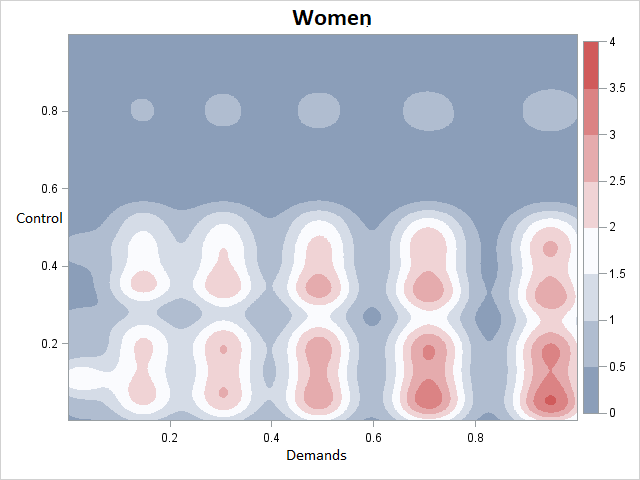
B)

**Supplementary Figure 1.** Kernel density plots over Job Exposure Matrix values of individuals for job demands and job control among a) all white-collar workers in the trade and retail industry (n=192,077) and b) women (n=85,356) and men (n=106,721).
